# Supplementary material for: Non-alcoholic fatty liver disease (NAFLD) and risk of hospitalization for Covid-19
Source: medRxiv. 2020 Sep 2:2020.09.01.20185850. Preprint. [Version 1] doi: 10.1101/2020.09.01.20185850 (PMC7480063; doi:10.1101/2020.09.01.20185850)
Supplement: Supplement 2020 [file 95280-2020.09.01.20185850-1.docx]

| **Table 1.** **Characteristics of individuals with a positive SARS-CoV-2 PCR who have NAFLD/NASH compared to those who do not.** | | | | |
| --- | --- | --- | --- | --- |
| n (%) | | No NAFLD/NASH  n = 6,429 | NAFLD/NASH  n = 373 | p-value |
| Age, median (IQR) | | 45.2 (27.4-66.5) | 56.1 (43.5-65.3) | <0.01 |
| Female | | 3604 (56.1) | 204 (52.9) | 0.24 |
| Race / Ethnicity | White | 2843 (53.2) | 154 (46.2) | <0.01 |
|  | Black | 1183 (22.1) | 53 (15.9) |  |
|  | Asian | 613 (11.5) | 68 (20.4) |  |
|  | Hispanic | 388 (7.3) | 38 (11.4) |  |
|  | Declined | 239 (4.5) | 12 (3.6) |  |
|  | Other | 75 (1.4) | 8 (2.4) |  |
| BMI, mean (SD), kg/m^2^ | | 29.8 (69.7) | 35.3 (8.2) | 0.15 |
| Co-morbidities | | | | |
| Obesity | | 3936 (61.2) | 305 (81.8) | <0.01 |
| Past Bariatric Surgery | | 57 (0.9) | 16 (4.5) | <0.01 |
| Elixhauser, mean (SD) | | 2.1 (3.0) | 6.0 (4.2) | <0.01 |
| Hypertension | | 2101 (33.2) | 254 (70.8) | <0.01 |
| Type 2 Diabetes | | 960 (15.2) | 170 (47.4) | <0.01 |
| Alcohol use disorder | | 243 (3.8) | 47 (13.1) | <0.01 |
| Home medications | | | | |
| Amiodarone | | 17 (0.3) | 3 (0.8) | 0.05 |
| Methotrexate | | 21 (0.3) | 3 (0.8) | 0.11 |
| Ca channel blocker | | 193 (3.0) | 28 (7.8) | <0.01 |
| Home oral steroid | | 226 (3.5) | 34 (9.5) | <0.01 |
| Metformin | | 141 (2.2) | 36 (10.0) | <0.01 |
| GLP-1 receptor agonist | | 50 (0.8) | 22 (5.9) | <0.01 |
| Outcomes from Covid-19 | | | | |
| Mortality | | 102 (1.6) | 14 (3.9) | <0.01 |
| Hospitalization | | 782 (12.2) | 148 (39.7) | <0.01 |
| ICU admission | | 340 (5.3) | 88 (24.5) | <0.01 |
| Abbreviations: NAFLD=non-alcoholic fatty liver disease. NASH=non-alcoholic steatohepatitis. PCR=polymerase chain reaction. IQR=inter-quartile range. BMI=body mass index. ICU=intensive care unit. SD=standard deviation | | | | |

| **Table 2a.** **Odds of admission for Covid-19 among those with +SARS-CoV-2 PCR, assessing demographic and clinical characteristics by logistic regression.^a^** | | | | |
| --- | --- | --- | --- | --- |
|  | | OR (95% CI) | | p-value |
| **Demographic characteristics** | | | | |
| Age | | 1.03 (1.02-1.04) | | <0.01 |
| Gender | | | | |
| Female | | Ref | |  |
| Male | | 1.28 (1.08-1.51) | | <0.01 |
| *Sub-Group Analysis* | | **- NAFLD/NASH** | **+ NAFLD/NASH** |  |
|  | Female | Ref | 1.71 (1.17-2.49) | 0.01 |
|  | Male | Ref | 2.48 (1.68-3.65) | <0.01 |
| Race/Ethnicity | | | | |
|  | White | Ref | |  |
|  | Black | 1.52 (1.19-1.95) | | <0.01 |
|  | Asian | 1.96 (1.43-2.70) | | <0.01 |
|  | Hispanic | 3.06 (2.15-4.36) | | <0.01 |
|  | Other | 1.278 (0.91-3.51) | | 0.09 |
| *Sub-Group Analysis* | | **- NAFLD/NASH** | **+ NAFLD/NASH** |  |
|  | White | Ref | 1.8 (1.15-2.69) | 0.01 |
|  | Black | Ref | 2.69 (1.40-5.16) | <0.01 |
|  | Asian | Ref | 2.11 (1.16-3.84) | 0.01 |
|  | Hispanic | Ref | 2.05 (0.95-4.43) | 0.07 |
| English-speaking | | 1.08 (0.86-1.35) | | 0.50 |
| **Co-morbidities** | | | | |
| Obesity | | 0.41 (0.34-0.49) | | <0.01 |
| Elixhauser comorbidity index | | 1.38 (1.33-1.41) | | <0.01 |
| Alcohol-use disorder | | 0.73 (0.52-0.10) | | 0.07 |
| **Home medications** | | | | |
| Amiodarone | | 0.13 (0.03-0.48) | | <0.01 |
| Oral Steroids | | 1.21 (0.85-1.72) | | 0.28 |
| Methotrexate | | 0.70 (0.23-2.12) | | 0.53 |
| Calcium Channel Blockers | | 0.80 (0.56, 1.15) | | 0.07 |
| **Table 2b. Secondary outcomes assessed, among patients with NAFLD/NASH** | | | | |
| Intensive Care Unit | | 1.70 (1.20-2.40) | | <0.01 |
| Intubation | | 1.98 (1.28-3.06) | | <0.01 |
| In-hospital mortality | | 0.99 (0.54-1.77) | | 0.94 |
| ^a^Adjusted for age, sex, obesity, ethnicity, NAFLD/NASH, alcohol abuse, Elixhauser comorbidity index, and home medications (amiodarone, methotrexate, oral steroids, or calcium channel blockers). | | | | |
